# Ampelopsin Confers Endurance and Rehabilitation Mechanisms in *Glycine max* cv. Sowonkong under Multiple Abiotic Stresses

**DOI:** 10.3390/ijms222010943

**Published:** 2021-10-10

**Authors:** Elham Ahmed Kazerooni, Abdullah Mohammed Al-Sadi, Il-Doo Kim, Muhammad Imran, In-Jung Lee

**Affiliations:** 1 Department of Applied Biosciences, Kyungpook National University, Daegu 41566, Korea; elham.ghasemi.k@gmail.com (E.A.K.); ildookim@hanmail.net (I.-D.K.); m.imran02@yahoo.com (M.I.); 2 Department of Plant Sciences, College of Agricultural and Marine Sciences, Sultan Qaboos University, P.O. Box 34, Al-Khod 123, Oman; alsadi@squ.edu.om

**Keywords:** soybean, salinity, heavy metal, ampelopsin, antioxidant enzymes, amino acids, fatty acid

## Abstract

The present investigation aims to perceive the effect of exogenous ampelopsin treatment on salinity and heavy metal damaged soybean seedlings (*Glycine max* L.) in terms of physiochemical and molecular responses. Screening of numerous ampelopsin concentrations (0, 0.1, 1, 5, 10 and 25 μM) on soybean seedling growth indicated that the 1 μM concentration displayed an increase in agronomic traits. The study also determined how ampelopsin application could recover salinity and heavy metal damaged plants. Soybean seedlings were irrigated with water, 1.5% NaCl or 3 mM chosen heavy metals for 12 days. Our results showed that the application of ampelopsin raised survival of the 45-day old salinity and heavy metal stressed soybean plants. The ampelopsin treated plants sustained high chlorophyll, protein, amino acid, fatty acid, salicylic acid, sugar, antioxidant activities and proline contents, and displayed low hydrogen peroxide, lipid metabolism, and abscisic acid contents under unfavorable status. A gene expression survey revealed that ampelopsin application led to the improved expression of *GmNAC109*, *GmFDL19*, *GmFAD3*, *GmAPX*, *GmWRKY12*, *GmWRKY142*, and *GmSAP16* genes, and reduced the expression of the *GmERF75* gene. This study suggests irrigation with ampelopsin can alleviate plant damage and improve plant yield under stress conditions, especially those including salinity and heavy metals.

## 1. Introduction

Plants have lived in inherently harsh environments ever since their emergence. A great range of physical and chemical stimuli, in the form of heat, frost, flood, drought, heavy metals and salinity, are hostile to them [1,2,3,4,5,6]. These stresses, unitedly named as abiotic stresses, cause serious peril to agriculture and the ecological system, accounting for huge crop yield loss around the world [7]. Salinity is the most tenacious stress, intensified by the accelerated salinization of cultivable land [8,9]. High salinity causes nutritional deficiency, oxidative injury, osmotic pressure and ion toxicity [10,11], which interfere with all the stages of the plant lifecycle, including seed germination, seedling formation, vegetative growth and fertility rate [12]. Over and above that, soil contamination is also predominant in various terrestrial ecosystems. It is affected by rapid industrialization, human activities, and modern agricultural systems. These processes can give rise to the dispersal of, for instance, heavy metals, which might influence food safety and ecosystem health [13,14]. Heavy metal accumulation poses the lowering of plant growth by detrimentally impacting several of the physiological and molecular functions of plants [15].

*Ampelopsis grossedentata* (Hand.-Mazz.) W.T. Wang (*Vitaceae*) is a flavonoid rich wild plant, which is historically consumed as herbal tea, and in traditional Chinese medicine, to treat pyretic fever, cough, common colds, pain, asthma, kidney injury, diabetes, swelling of the pharynx and larynx and jaundice hepatitis [16,17,18]. Ampelopsin (also termed Dihydromyricetin), the most common flavonoid compound, was first isolated from this plant by Kotake and Kubota [19]. Nowadays, ampelopsin is known for its pharmacological functions and potential human health benefits including antitumor activity, antibacterial and anti-inflammatory effects, antihypertension impacts, dermaprotective effect, neuroprotective and antioxidant effects [18,20]. Moreover, ampelopsin has been used as a successful hangover remedy and supplement to ease alcohol use disorders (AUDs) [21].

Soybean (*Glycine max* L.) is one of the most economically important food crops in the world. This crop is a vital source of vegetable oil and vegetable protein. The quality of soybean protein is greater than other plant proteins, and identical to animal protein [22]. Furthermore, it contains numerous unique nutriments, namely, isoflavone, saponin, and phytosterol [23,24]. Apart from its consumption, soybean oil and protein is being taken into consideration as a future source of fuel and alternative for plastics, respectively [25,26]. However, its production and yield is largely affected by various abiotic stresses enforced by environmental factors, including heavy metals, salinity, high temperature, ultraviolet radiation, and deficient or excessive water [27]. This legume is categorized as a fairly salt and heavy metal sensitive crop, and its yield has been negatively affected by these abiotic stresses [28,29]. Increasing salinity levels impose harmful effects on soybean growth, nodulation, agronomic traits, seed quantity and quality, and, therefore, reduces soybean yield [30,31,32].

Since various abiotic stresses commonly emerge in the field, it is becoming indispensable to equip crops with multiple stress endurance to ease the pressure of environmental changes and to fulfill the need of population growth. We speculated that ampelopsin could enhance heavy metal, along with salinity, endurance in soybean plants under deleterious environmental situations. Therefore, we explored, for the first time, whether the application of ampelopsin could promote the rehabilitation ability, and sustain the growth of, salinity and heavy metal stressed soybeans. We attempted to identify the suitable ampelopsin concentration that was effective toward salinity and heavy metal impaired plants. Then, we aimed to decipher how exogenous ampelopsin application influences the physiochemical attributes of salinity and heavy metal damaged plants by examining growth alterations, fatty acid contents, amino acid contents and antioxidant contents. Moreover, we evaluated the transcription patterns of various genes, including *GmWRKY12* and *GmWRKY142* [33,34], *GmNAC109* [35], *GmERF75* [36], *GmFDL19* [37], *GmSAP16* [38], *GmFAD3* [37], and *GmAPX* [39], to confirm our physiochemical findings.

## 2. Results

### 2.1. The Growth Performane of Soybean Seedlings under Varied Salt Concentrations

Irrigation with the minimum NaCl concentration (0.5%) caused no impressive change in relation to the control plants (Table S3; Figure S1). However, soybean plants irrigated with NaCl at 1.5% had a much reduced plant height (53.7%), root length (53.19%), stem diameter (40.63%), leaf length (51.63%), leaf width (59.62%), plant fresh weight (66.86%), plant dry weight (53.7%), root fresh weight (74.41%), root dry weight (86.67%), and leaf number (35.92%) (*p* < 0.05), with respect to these measurements in control plants (Table S3; Figure S1). Additionally, irrigation with the utmost concentration of NaCl (2.5%) led to an extreme mitigation in plant growth traits. Thereby, 1.5% NaCl was the ideal concentration appointed for usage in the upcoming survey. This concentration is also common in different parts of the world, especially in areas affected by soil salinity.

### 2.2. The Growth Performance of Stressed Soybean Seedlings under Different Ampelopsin Concentrations

We recorded a notable alleviation in the different plant growth parameters in salinity and heavy metal stressed plants, as compared to the control (Table S4). In contrast, when salinity and heavy metal stressed plants gained varied concentrations of AMP (0, 0.1, 1, 5, 10 and 25 μM), they typically demonstrated a mitigation of salt and heavy metal stresses (Table S4; Figures S2–S5). Treatment with a minimum concentration of AMP (0.1 μM), or with a maximum concentration of AMP (25 μM), exhibited no outstanding alteration compared with salinity and heavy metal stressed plants. Albeit, salinity and heavy metal-stressed plants at an AMP concentration of 1 μM displayed increased plant growth characteristics. For instance, improved plant height (44.44%), root length (43.16%), stem diameter (22.73%), leaf length (53.72%), leaf width (37.26%), and chlorophyll content (39.32%) (*p* < 0.05) were recorded in AMP treated stressed plants, in contrast to salinity stressed plants alone (Table S4; Figures S2–S5). These outcomes showed that 1 μM AMP diminished the repressing impact of salinity and heavy metal stresses on plant growth.

### 2.3. Recovery Influence of Exogenous AMP on Stressed Soybean Seedlings

#### 2.3.1. Changes in Plant Growth Attributes

**Figure 1:**
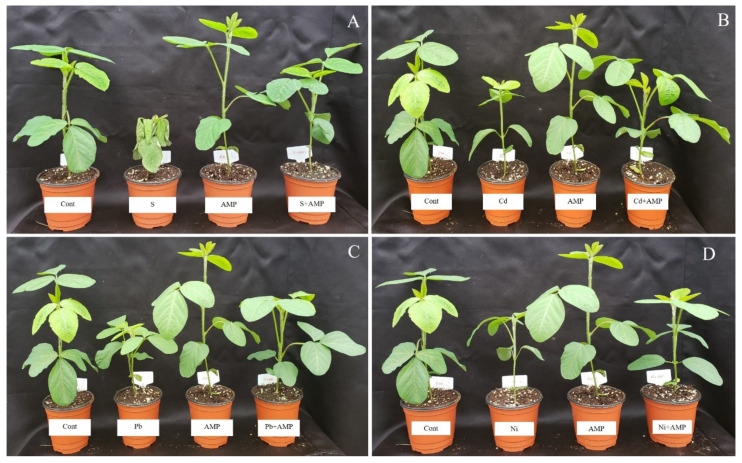
Effects of ampelopsin application on soybean plant growth under normal and stress conditions after 12 days of treatment (12DAT; (**A–D**)). Treatments: Cont (control), AMP (1 μM ampelopsin), S (1.5% sodium chloride), AMP (1 μM ampelopsin) + S (1.5% sodium chloride), Cd (3 mM cadmium), 1 μM AMP (ampelopsin) + Cd (3 mM cadmium), Pb (3 mM lead), AMP (1 μM ampelopsin) + Pb (3 mM lead), Ni (3 mM nickel), and AMP (1 μM ampelopsin) + Ni (3 mM nickel). Values show the means ± SE (*n* = 5) and significant differences at *p* < 0.05 (Tukey test).

**Figure 2:**
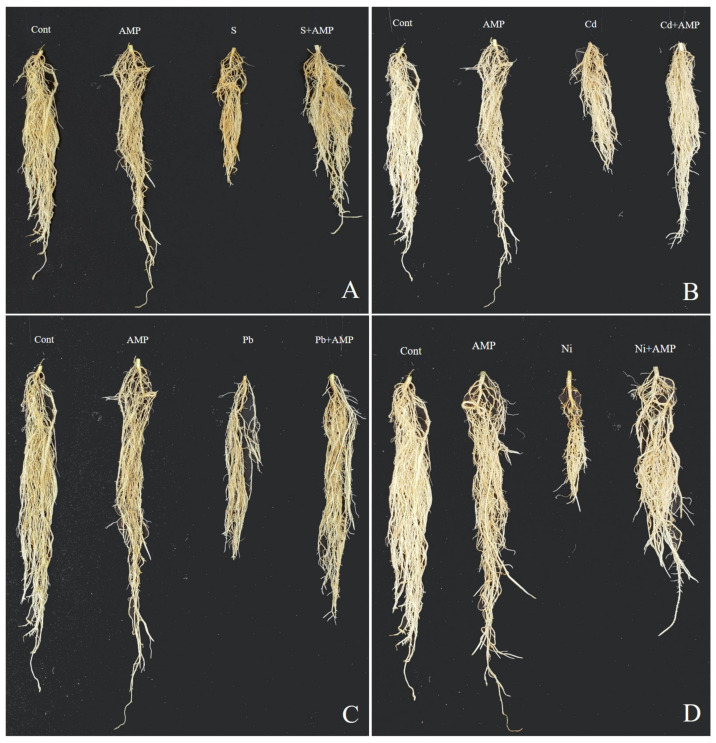
Effects of ampelopsin application on soybean plant roots under normal and stress conditions after 12 days of treatment (12DAT; (**A–D**)). Treatments: Cont (control), AMP (1 μM ampelopsin), S (1.5% sodium chloride), AMP (1 μM ampelopsin) + S (1.5% sodium chloride), Cd (3 mM cadmium), 1 μM AMP (ampelopsin) + Cd (3 mM cadmium), Pb (3 mM lead), AMP (1 μM ampelopsin) + Pb (3 mM lead), Ni (3 mM nickel), and AMP (1 μM ampelopsin) + Ni (3 mM nickel). Values show the means ± SE (*n* = 5) and significant differences at *p* < 0.05 (Tukey test).

The impact of the AMP on the growth of soybean seedlings under salinity, Cd, Pb, and Ni stress conditions, and without stress, was investigated in pot trials (Figure 1 and Figure 2). The detrimental impacts of these abiotic stresses negatively impacted growth parameters, which included plant height, stem diameter, leaf area (length/width), plant weight (fresh/dry), root weight (fresh/dry) and number of leaves, with reference to corresponding parameters in unstressed and untreated soybean plants (Table 1). In contrast, these growth parameters were increased in AMP treated plants under stress conditions. Particularly, plant height was promoted by 53.53%, 26.32%, 35.17% and 36.28% in the salt, Cd, Pb and Ni treatments, respectively, in contrast with the corresponding heights of untreated stressed plants (*p* < 0.05). Likewise, in AMP treated plants, root length was increased by 36.17%, 43.04%, 33.07%, and 52.98% in the salt, Cd, Pb and Ni treatments, respectively, in comparison to the root length of the control, stressed group of plants (Table 1).

#### 2.3.2. Chlorophyll and Carotenoid Content

Photosynthetic pigment value was investigated in soybean plants with or without stress conditions. In the current survey, our values revealed that Chla, Chlb, and carotenoid values were higher in AMP treated stressed plants as compared to the untreated stressed plants. Likewise, greater values of total chlorophyll content were recorded in AMP treated stressed plants, with respect to the control stressed plants (Table 2). A reduction in total chlorophyll content was noticed in salt, Cd, Pb and Ni stressed plants (41.07%, 11.18%, 5.97%, and 4.59%, respectively), compared to control plants. In contrast, the AMP application was efficient (*p* < 0.05) and contributed to a 35.18%, 7.66%, 4.07%, and 4.34% enhancement in total chlorophyll content under salt, Cd, Pb and Ni stress conditions, respectively, as compared to the control stressed plants (Table 2).

#### 2.3.3. Phytohormone (ABA and SA) Regulation

**Figure 3:**
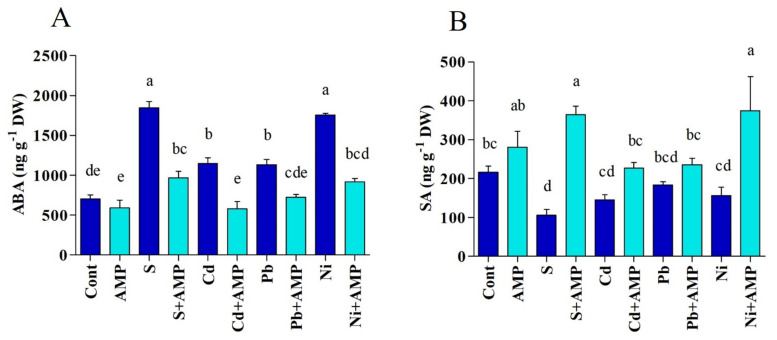
(**A**) ABA and (**B**) SA content in leaves of soybean grown under normal and stress conditions and treated with ampelopsin for 12 days (12DAT). Treatments: Cont (control), AMP (1 μM ampelopsin), S (1.5 % sodium chloride), AMP (1 μM ampelopsin) + S (1.5 % sodium chloride), Cd (3 mM cadmium), 1 μM AMP (ampelopsin) + Cd (3 mM cadmium), Pb (3 mM lead), AMP (1 μM ampelopsin) + Pb (3 mM lead), Ni (3 mM nickel), and AMP (1 μM ampelopsin) + Ni (3 mM nickel). Values show the means ± SE (*n* = 5) and significant differences at *p* < 0.05 (Tukey test). Bars with different lowercase letters are significantly different from each other.

Variation in the content of endogenous ABA and SA are presented in Figure 3A,B. Salinity and heavy metal stresses significantly excreted the accumulations of ABA in soybean seedlings. However, AMP application diminished ABA levels in soybean plants, with respect to the levels in control plants not exposed to abiotic stresses. Following exposure to salinity, Cd, Pb and Ni stresses, AMP treated plants displayed a meaningfully lowered content of ABA, i.e., 47.62%, 49.28%, 36.18%, and 47.67%, respectively, as compared to the content of untreated stressed plants (Figure 3A).

By comparison with stressed plants, untreated plants revealed a decline in SA concentrations, which were alleviated by 51.10% with salinity and 32.60% with Cd, 14.95% with Pb, and 27.75% with Ni, with respect to the SA concentrations in normal plants. Soybean seedlings treated with AMP for 12 days, meanwhile, depicted noticeable enhancement, 71.73%, 35.91%, 21.76%, and 58.28% in SA content under salt, Cd, Pb, and Ni stresses, respectively, in comparison with untreated stressed plants (Figure 3B). In fact, our results implied that AMP application led to improved SA content in soybean seedlings under both normal and stress conditions.

#### 2.3.4. Free Amino Acid Content

We quantified eighteen amino acids in soybean seedlings exposed to various treatments (Table 2). Abiotic stresses caused marked increases in amino acid content in soybean seedlings over 12 days. Proline content increased by 16.57%, 15.54%, 17.03%, and 14.75% in salinity, Cd, Pb, and Ni stressed plants, respectively, as compared to the seedlings under ordinary conditions. Likewise, plants treated with AMP had increased proline content. Moreover, 12 days after the application of AMP on impaired plants, asparagine was highest in salt, Cd, and Pb stressed plants (but not Ni stressed plants), while cystine and methionine were lowest in soybean seedlings exposed to normal, as well as stress, conditions (Table 2). These results show that AMP application generally increased amino acid content in soybean plants, with or without stress.

#### 2.3.5. Total Protein and Sugar Content

**Figure 4:**
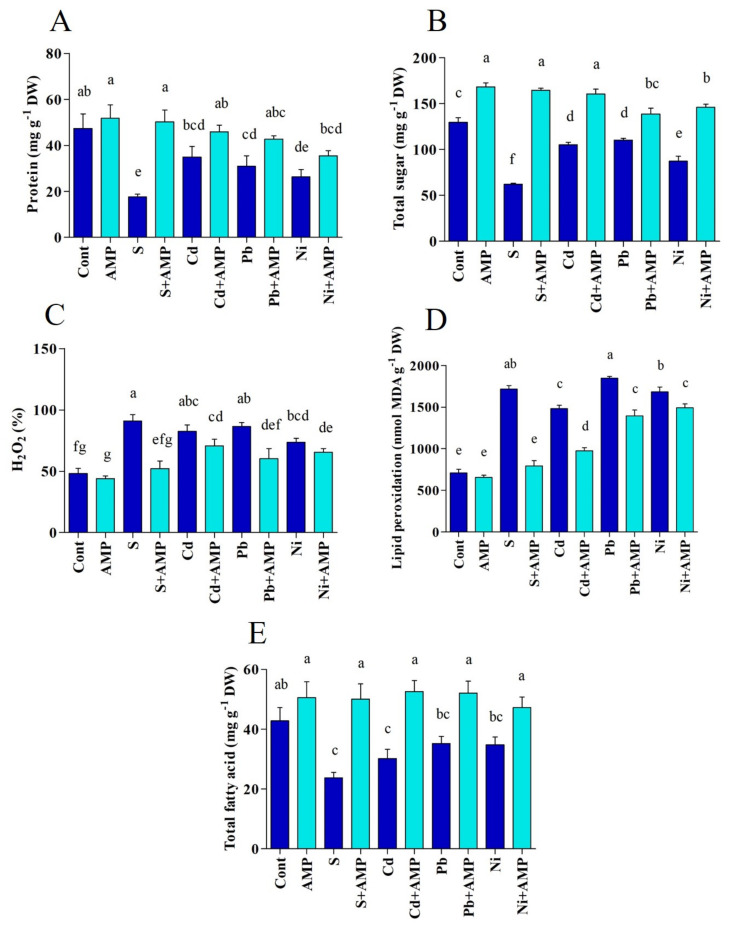
(**A**) Protein, (**B**) Sugar, (**C**) H_2_O_2_, and (**D**) MDA, (**E**) total fatty acid content in leaves of soybean grown under normal and stress conditions and treated with ampelopsin for 12 days (12DAT). Treatments: Cont (control), AMP (1 μM ampelopsin), S (1.5% sodium chloride), AMP (1 μM ampelopsin) + S (1.5% sodium chloride), Cd (3 mM cadmium), 1 μM AMP (ampelopsin) + Cd (3 mM cadmium), Pb (3 mM lead), AMP (1 μM ampelopsin) + Pb (3 mM lead), Ni (3 mM nickel), and AMP (1 μM ampelopsin) + Ni (3 mM nickel). Values show the means ± SE (*n* = 5) and significant differences at *p* < 0.05 (Tukey test). Bars with different lowercase letters are significantly different from each other.

Protein content increased by 8.47% upon AMP application under ordinary conditions, as compared with untreated plants. While it was decreased by 62.85%, 26.18%, 34.73%, and 44.48% under salinity, Cd, Pb and Ni stresses (Figure 4A). Under stress conditions, AMP treated stressed plants had higher protein contents than stressed plants, and were very similar to levels seen in unstressed controls (salt, 64.95%; Cd, 23.76%; Pb, 30.90%; Ni, 25.82%).

A considerable drop in sugar content was noticed in soybean plants after exposure to stress conditions (Figure 4B). This drop was mainly seen in response to salt (51.97% drop), but drops were also seen in Cd (18.68%), Pb (14.75%), and Ni (32.54%) stressed plants (Figure 4B). AMP application contributed to a rise in sugar content by 62.20%, 34.41%, 20.38%, and 40.25% under salt, Cd, Pb and Ni stresses conditions, with respect to the sugar content of stressed plants alone (Figure 4B).

#### 2.3.6. H_2_O_2_, MDA and Fatty Acid Content

Abiotic stresses created noticeable alterations in H_2_O_2_ content in soybean plants (Figure 4C). H_2_O_2_ content was elevated by 46.87%, 41.51%, 44.18%, and 34.37% under salinity, Cd, Pb, and Ni stresses, respectively, as compared to the H_2_O_2_ content in unstressed plants. However, the application of AMP was efficient in diminishing H_2_O_2_ content in stressed plants; utmost mitigations of 42.53%, 14.27%, 30.19%, and 10.87% in H_2_O_2_ content were documented in AMP treated plants under salinity, Cd, Pb and Ni stresses, respectively (*p* < 0.05).

As illustrated in Figure 4D, stress conditions promoted malondialdehyde (MDA) generation in untreated soybean plants. AMP treatment ameliorated MDA evolution under salt and, to a lesser extent, cadmium stress but had little effect in lead and nickel stressed plants (*p* < 0.05).

Total fatty acid content in soybean seedlings decreased in response to salt (44.62%), Cd (29.43%), Pb (17.75%), and Ni (18.69%) stresses, as compared to the fatty acid content of plants under nonstress conditions (Figure 4E). However, AMP treated plants exhibited higher fatty acid contents under all stresses, and even that of the unstressed control.

#### 2.3.7. Antioxidant Content

**Figure 5:**
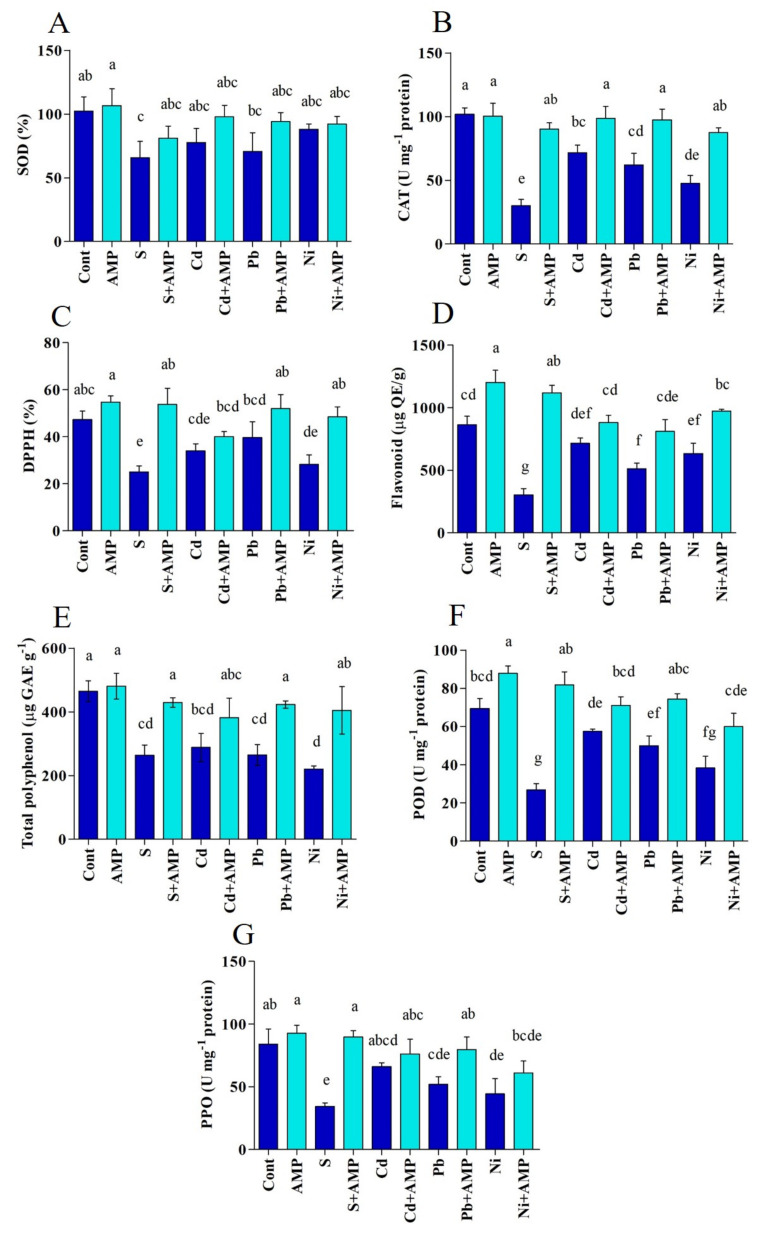
Antioxidant content (SOD), (**A**); CAT, (**B**); DPPH, (**C)**; flavonoids, (**D**); total polyphenol, (**E**); POD, (**F**); and PPO, (**G**) of soybean leaves grown under normal and stress conditions and treated with ampelopsin for 12 days (12DAT). Treatments: Cont (control), AMP (1 μM ampelopsin), S (1.5% sodium chloride), AMP (1 μM ampelopsin) + S (1.5% sodium chloride), Cd (3 mM cadmium), 1 μM AMP (ampelopsin) + Cd (3 mM cadmium), Pb (3 mM lead), AMP (1 μM ampelopsin) + Pb (3 mM lead), Ni (3 mM nickel), and AMP (1 μM ampelopsin) + Ni (3 mM nickel). Values show the means ± SE (*n* = 5) and significant differences at *p* < 0.05 (Tukey test). Bars with different lowercase letters are significantly different from each other.

Antioxidant activities were evaluated in soybean plants under normal as well as stress conditions. Overall, we observed a reduction in enzymatic and nonenzymatic antioxidant functions (SOD, CAT, DPPH, flavonoid, total polyphenol, POD and PPO) in soybean plants in response to stress conditions. On the other hand, AMP application evidently raised antioxidant activities under these stress conditions (Figure 5A–G). For instance, SOD and CAT activity was higher in AMP treated plants affected by the salinity (SOD, 22.86%; CAT, 66.53%), Cd (SOD, 20.63%; CAT, 27.39%), Pb (SOD, 27.13%; CAT, 36.16%), and Ni (SOD, 6.38%; CAT, 45.60%) stresses, as compared to untreated stressed plants (Figure 5A,B).

### 2.4. Effect of Ampelopsin (AMP) Application on the Expression of Salinity and Heavy Metal Responsive Genes

The transcription pattern of selected abiotic stress responsive genes was assessed in soybean seedlings. Altogether, eight genes were examined for their alteration in expression in soybean plants under AMP application and chosen abiotic stresses.

#### 2.4.1. Transcription Factor WRKY

**Figure 6:**
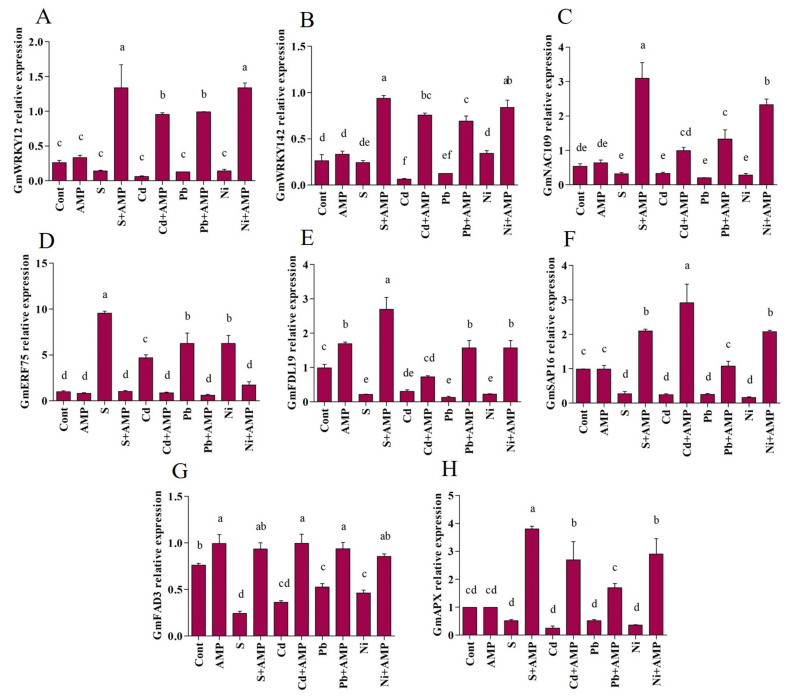
Real time expression analysis of WRKYs GmWRKY12, (**A**); and GmWRKY142, (**B**); GmNAC109, (**C**); GmERF75, (**D**); GmFDL19, (**E**); GmSAP16, (**F**); GmFAD3, (**G**); and GmAPX (**H**) in leaves of soybean grown under normal and stress conditions and treated with ampelopsin after 12 days (12DAT). Treatment: Cont (control), AMP (1 μM ampelopsin), S (1.5% sodium chloride), AMP (1 μM ampelopsin) + S (1.5% sodium chloride), Cd (3 mM cadmium), 1 μM AMP (ampelopsin) + Cd (3 mM cadmium), Pb (3 mM lead), AMP (1 μM ampelopsin) + Pb (3 mM lead), Ni (3 mM nickel), and AMP (1 μM ampelopsin) + Ni (3 mM nickel). Values show the means ± SE (*n* = 5) and significant differences at *p* < 0.05 (Tukey test). Bars with different lowercase letters are significantly different from each other.

This study characterized two genes (*GmWRKY12* and GmWRKY142) in soybean plants. Under AMP application and abiotic stress, these genes represented contrasting responses in soybean seedlings. A decrease in *GmWRKY12* expression levels was depicted in salt and heavy metal stressed plants, with respect to expression in unstressed plants. However, among salt, Cd, Pb, and Ni stressed plants, the AMP application enhanced expression by 89.28%, 93.49%, 87.35%, and 89.28%, respectively, contrasted with that in untreated plants (Figure 6A). Furthermore, these stresses reduced *GmWRKY142* expression in untreated stressed plants, as compared with treated control plants. A higher *GmWRKY142* expression level was demonstrated in AMP treated salt and heavy metal-stressed plants, with respect to the expression in untreated stressed plants (Figure 6B).

#### 2.4.2. Transcription Factor NAC

The quantitative expression of *GmNAC109* genes in soybean seedlings under varied treatments is illustrated in Figure 6C. Exposure to chosen stresses declined *NAC* gene expression in soybean plants, while AMP treatment improved the expression of this gene in soybean plants. For example, AMP application promoted *GmNAC109* gene expression by roughly 89.55%, 66.64%, 84.31%, and 87.61% under salinity, Cd, Pb and Ni stresses, contrasted with the relative expressions in stressed plants alone.

#### 2.4.3. Ethylene Responsive Element Binding Factor (ERF75)

The impacts of abiotic stresses along with AMP application on Ethylene responsive factor 75 (*ERF75*) was investigated in soybean seedlings via the alteration in the *ERF75* gene expression (*GmERF75*) (Figure 6D). Under unstressed conditions, slight differences were noticed in the expression of the *GmERF75* gene in control and AMP treated plants; in contrast, elevated *GmERF75* expression was observed in stressed plants. AMP treated plants revealed a decline in *GmERF75* expression contrasted with untreated stressed plants (89.35% under salinity, 81.17% under Cd, 90.07% under Pb, and 72.50% under Ni stress conditions).

#### 2.4.4. Basic Leucine Zipper Transcription Factor (FDL19)

The relative expression of the transcription factor *FDL19* (*GmFDL19*) was also studied. A decrease in *GmFDL19* expression levels was detected in salinity (78.20%), Cd (69.37%), Pb (87.04%), and Ni (76.97%) stressed plants, in comparison with the expression pattern recognized in normal plants. However, AMP treated stressed plants demonstrated an increase in gene expression levels, with respect to the expression recorded in untreated stressed plants. *GmFDL19* levels in stressed soybean plants were increased by 91.96% under salinity, 58.26% under Cd, 91.82% under Pb, and 85.46% under Ni in AMP treated stressed plants in comparison to untreated stressed plants (Figure 6E).

#### 2.4.5. Stress Associated Protein (SAP16)

As depicted in Figure 6F, salinity, Cd, Pb and Ni stressed plants exhibited a 72.27%, 75.04%, 73.36%, and 83.44% reduction, respectively, in *GmSAP16* expression, compared with the expression perceived in normal plants. On the other hand, enhanced expression of *GmSAP16* was noticed in AMP treated plants subjected to abiotic stresses. AMP treatment elevated the *GmSAP16* expression by 86.76% under salinity, 91.50% under Cd, 75.48% under Pb, and 92.09% under Ni stresses, compared to the untreated stressed plants (Figure 6F).

#### 2.4.6. Fatty Acid Desaturase 3 (FAD3)

Reduced *GmFAD3* expression was observed in soybean seedlings subjected to abiotic stress. The *GmFAD3* expression level reduced by 68.06% (salt), 52.47% (Cd), 31.02% (Pb), and 39.34% (Ni) compared to the unstressed control plants (Figure 6G). However, AMP treated soybean plants showed increased GmFAD3 expression under stress conditions. Salt, Cd, Pb, and Ni stressed plants exhibited 73.95%, 63.61%, 43.95%, and 45.96% higher *GmFAD3* expression compared to the untreated stressed plants.

#### 2.4.7. Enzymatic ROS Scavenging, Ascorbate Peroxidase (GmAPX)

The *GmAPX* expression level in soybean seedlings under abiotic stresses and AMP application is depicted in Figure 6H. A decrease in the *GmAPX* expression level was detected in salt, Cd, Pb, and Ni-stressed plants by 48.27%, 74.29%, 48.27%, and 64.21%, respectively, as compared to the unstressed plants. Contrarily, the application of AMP increased the *GmAPX* expression content in salt (86.48%), Cd (90.52%), Pb (69.68%), and Ni (87.75%) stressed soybean plants, in comparison with the corresponding untreated stressed plants.

## 3. Discussion

We have explored the impacts of ampelopsin (AMP) on soybean seedlings. 1 μM AMP treated seedlings seemed to be healthier, and we noticed that AMP, when irrigated into soil, improves plant growth and development. Furthermore, it promotes salt and heavy metal tolerance, as observed from the enhanced growth parameters. Our outcomes illustrated that unstressed and stressed seedlings treated with AMP sustain greater height, root length, leaf area, etc., in contrast to stressed plants in the absence of AMP. We perceived rises in chlorophyll and carotenoid values in the AMP treated plants under both stress and normal conditions. These functions are probably accomplished via amelioration processes connected with photosynthesis and the other metabolisms. These findings present a novel approach for boosting the production of soybean and, perhaps, other crops regularly used in agriculture.

The plant specific NAC (an abbreviation for NAC, ATAF and CUC) superfamily includes one of the biggest families of transcription factors [40]. Several NAC members have been practically distinguished in floral formation [41], apical meristem development [42], lateral root development [43,44,45], growth hormone signaling [43,46], and leaf senescence [47]. Various NAC genes are involved in plant developmental processes and reaction to abiotic stresses, namely, dehydration, flood, drought, salinity, and cold [48,49,50,51]. We observed the enhanced expression of *GmNAC109* in AMP treated seedlings exposed to abiotic stresses. Nguyen, Hoang, Nguyen, Binh, Watanabe, Thao and Tran [35] reported that enhanced *GmNAC109* led to enhanced drought tolerance, decreased endogenous hydrogen peroxide and stronger superoxide dismutase and catalase activities. Overall, our results revealed that the overexpression of *GmNAC109* in AMP treated plants enabled them to cope with adverse condition.

Phytohormones play pivotal roles in plant development and growth regulation, and can improve plant response to environmental stimuli [52]. Salicylic acid (SA) is engaged in the modulation of essential plant physiological processes, including photosynthesis, antioxidant defense system and proline metabolism, and contribute to plant protection against abiotic stresses [53]. Former reports have shown the effect of abiotic stress on phytohormone performance, involving that of SA and abscisic acid (ABA) [54,55]. It has been demonstrated that salicylic acid improves plant endurance of abiotic stresses, including salinity and heavy metal, by inducing various genes [56,57,58]. Salicylic acid was reported to improve salt tolerance by inducing the transcription patterns of antioxidant genes [59]. Yasuda et al. [60] demonstrated that incompatible crosstalk occurs within SA and ABA pathways. The current survey findings imply that abiotic stresses reduce SA levels but increase ABA levels, which is in harmony with earlier reports [61,62,63,64]. Our findings show that applying AMP improves the ability of soybean seedlings to resist stressful environment by reducing the ABA contents and boosting the SA content.

WRKY proteins, the superfamily of transcription factors, participate in numerous developmental and physiological functions, including senescence, plant immune response, development, pathogen defense, seed development, democracy, and germination [65,66,67,68,69,70,71]. Several studies have confirmed that WRKY proteins are involved in abiotic stresses, including salt, water deficit, and low and high temperature [72,73,74,75]. The examination of WRKYs expression in the present survey disclosed their reduced expression under abiotic stress conditions. Varied WRKY genes have been proven to play a role in ABA and SA pathways [76,77]. One earlier investigation revealed that ABA regulates the transcripts of some WRKYs in a negative or positive way [78]. We noticed that *GmWRKY12* and *GmWRKY142* transcript patterns were lower in soybean plants subjected to chosen stresses in the presence of ABA. Conversely, once ABA levels were diminished in AMP treated stressed plants, the expression pattern of *GmWRKY12* and GmWRKY142 escalated. These outcomes prove that ABA negatively modulates the expression of *WRKY12* and *WRKY142* in soybean plants. Previous studies showed that the enhanced expression of *GmWRKY12* and *GmWRKY142* led to improved salt and cadmium tolerance, augmented proline content and alleviated malondialdehyde content under salt and cadmium treatment in soybean seedlings [33,34]. In sum, our findings tied to *GmWRKY12*, *GmWRKY142*, ABA, and SA quantities in AMP treated seedlings enable us to assume that AMP assists stressed plants to deal with numerous abiotic stresses.

Increased evidence has demonstrated that members of the stress associated proteins (SAPs) gene family is engaged in the modulation of environmental stress responses. It has been proven that the SAP gene family contribute to endurance of numerous abiotic stresses in plants [79,80,81,82,83]. Mukhopadhyay, Vij and Tyagi [79] reported that *OsISAP1* overexpression conferred cold, aridity, and salt stress tolerance in tobacco seedlings. Similarly, *AtSAP13* overexpression upregulation and vigorous tolerance in reaction to heavy metal, salt and drought stresses [84]. Dixit and Dhankher [82] demonstrated that the overexpression of *AtSAP10* led to improved endurance to heat and heavy metals, such as Ni, Mn, and Zn. In the current survey, both biochemical and molecular analyses depicted that AMP treated stressed plants displayed improved protein content at the end of the assay. We have shown that *GmSAP16* is differentially regulated in AMP treated plant under stress conditions. Our outcomes disclosed that the overexpression of *GmSAP16* provides endurance to salinity and toxic heavy metals.

Plants exposed to environmental stresses are prone to oxidative stress because of reactive oxygen species (ROS) formation, which, at larger amounts, can induce oxidative damage, destroy membrane lipid, hinder metabolic functions, activate program cell death, and deactivate enzymes [85]. Considering the current survey, we noticed that H_2_O_2_ and MDA contents were obviously higher in stressed plants, which could be due to the imbalance rate of ROS production and removal [86]. AMP application, however, apparently attenuated the raised H_2_O_2_ and MDA contents in stressed plants toward the end of the investigation. Thus, AMP might restrain the generation of ROS and, therefore, impede oxidative based plasma membrane impairment under unfavorable conditions [87,88,89,90].

Land plants are dwelling in a rough environment that enforces extremely varied stresses on their health and productivity. Hence, they have developed intricate strategies to escape or abide the detrimental effects. Fatty acids are coming into center of attention as one of the common defense mechanisms against several biotic and abiotic stresses [91,92]. Previous studies have shown that fatty acids modulate ROS levels by particular impacts on ROS-generating enzymes [93]. The results revealed that salinity and heavy metal stresses influence the fatty acid content. We observed lower fatty acid contents in soybean seedlings subjected to abiotic stresses. In contrast, AMP treated seedlings were less affected by these stresses and showed a significantly higher level of fatty acids under stress conditions. Moreover, we observed the expression of fatty acid desaturase 3 (FAD3) enzymes, which catalyze the conversion of linoleic acid to α-linolenic acid. Recent studies showed that fatty acid overexpression improved cold, drought and osmotic stress endurance in tobacco, soybean and tomato plants [94,95,96]. Subsequently, from these findings, we can come to the conclusion that AMP application may cause the activation of phospholipases, as well as phospholipid derived molecules, which are involved in plant defense systems [97]. 

The ethylene responsive factor (ERF) family contains a wide range of elements with multifarious functions, which regulate physiological, developmental and environmental stimuli responses [36,98]. ERF modulates various plant responses, such as light acclimation, flower pedicel abscission, leaf senescence, cell proliferation, bud outgrowth, and shoot branching [99,100,101,102]. Moreover, ERF plays a role as an important regulator of osmotic and hypoxic stress responses, and maintains hydrogen peroxide homeostasis in plants [36]. Our results depicted enhanced expression of *GmERF75* in salinity and heavy metal stressed seedlings, which was consistent with former findings. Previous reports showed the rapid induction of ERF under various stresses, including salt, drought, pathogen infection, wounding and temperature [36,98,103,104]. The expression level of *GmERF75* is rapidly downregulated in seedlings upon AMP application, which proves the stress alleviation effect of AMP.

To survive, plants have developed complicated tactics to react to stress via changes at physiological and molecular levels [105]. Plants mainly combat against oxidative damage and eliminate excessive ROS augmentation through endogenous defensive procedures that engage antioxidant functions [106,107]. In the present survey, antioxidants’ function decreased in stressed plants, though this function was elevated following AMP application in these abiotic stressed plants. Previous studies indicated that abiotic stresses could either induce or impede the expression of antioxidant enzymes [39,108,109,110]. This increase in antioxidant activity implies that AMP enhances the capability to scavenge excessive ROS, mitigates oxidative damage, and improves tolerance to oxidative stress, which provides the protection of photosynthetic processes.

The plant basic leucine zipper (bZIP) family takes part in developmental processes and responds to abiotic stresses comprising salinity and drought [58,111,112]. In the present study, the attenuated gene expression of *GmFDL19*, a bZIP transcription factor, was detected in stressed plants, which presumably led to plant retardation and reduced abiotic stress tolerance in soybean seedlings. It has been shown that the overexpression of *GmFDL19* promotes salt and drought endurance in soybean plants [37]. AMP treated stressed plants presented elevated *GmFDL19* gene expression, which caused improved plant height, dry weight, antioxidant enzyme activity, chlorophyll content and reduced MDA content. This was in line with a previous report [37]. Our outcomes suggest that AMP application alleviates the expression of *GmFDL19* and boosts abiotic stress endurance in soybean seedlings.

Sugar molecules are the most critical regulators that promote various physiological processes, such as protein synthesis, lipid metabolism, photosynthesis, and osmotic homeostasis [113]. Unfavorable environmental conditions can diminish leaf sugar content and, consequently, induce physiochemical alterations [114]. It has been confirmed that the augmentation of soluble sugars promotes growth and abiotic stress endurance in plants, since the soluble sugars or their byproducts act as osmotically active molecules under stressful situations [115]. In the present study, a distinct accumulation of sugars was detected in soybean seedlings treated with AMP under normal and stress conditions. Sugars act as metabolic resources, stabilize membranes, and they regulate numerous procedures connected with plant performance under deleterious conditions [113,116]. It has been demonstrated that sugar augmentation also improves proline content under stress conditions [117]. Considering the current survey, AMP treatment caused greater sugar augmentation, which probably performed as an osmoprotectant to regulate osmotic adjustments, protect membrane and scavenge ROS under various stress conditions.

Amino acids take part in the synthesis of various plant products which regulate plant responses to unfavorable growth conditions [118]. In our survey, amino acid content escalated in soybean seedlings under abiotic stresses. This augmentation of amino acids may be involved in osmotic acclimatization, free radical scavenging and protein maintenance [119]. Earlier inspections have stated that amino acid content escalates in plants under stress [118,120]. The application of AMP recovered amino acid value in stressed seedlings throughout the restoration time. A quick rise in proline amount was viewed in stressed soybean seedlings. This is in harmony with the discoveries of former research on different kinds of plants [121,122]. The augmentation of proline, which serves as a reactive oxygen species scavenger, could prove to be a strategy through which plants withstand abiotic stress [123,124,125,126]. Assuredly, enhanced proline content has been closely tied to stabilizing membrane and protein structure, minimizing cell damage, and enhancing plant survival under environmental changes [62,127]. Furthermore, proline not only functions as an osmotolerant, but also acts as a nutritional reservoir that can be utilized during the restoration phase to assist plants combat environmental predicaments [128,129]. In this report, AMP utilization resulted in increased amino acids, especially proline content in stressed seedlings; this AMP induced proline enhancement could express an adaptive procedure that promotes osmotic acclimatization and reduces the detrimental effect of salinity and heavy metal stresses. 

## 4. Materials and Methods

### 4.1. Selection of Appropriate Concentrations of Salt, Cadmium, Lead and Nickel

Soybean seeds were provided by the Agricultural Research and Extension Services (Daegu, Gyeongsangbuk-do, Republic of Korea). Seeds of equal size/same color were picked and submerged in 70% ethanol and 2.5% sodium hypochlorite and checked for the effectiveness of the disinfection procedure and vitality [130,131]. Seeds (one seed/pot) were sown in pot trays (28 cm × 54 cm) filled with horticultural soil (Shinsung Mineral Co., Ltd., Daegu, Chungcheongbuk-do, Republic of Korea). Afterwards, trays were placed in a climate chamber and plants were grown under 16/8 h day/night regime (250 μmol photons m^−2^ s^−1^ irradiance, 65% relative humidity), at 26 ± 2 °C, and irrigated daily. After three weeks, seedlings with identical sizes were shifted to pots (10 × 10 cm) and received varied treatments. All soybean seedlings were arbitrarily separated into two groups as follows: (i) control, irrigated with distilled water (50 mL/pot), and (ii) salinity treatment, irrigated with NaCl (0.5, 1, 1.5 and 2.5%) (50 mL/pot). Each treatment included five replicates. Plants treated with each concentration were evaluated for varied morphological characteristics after day 7 (7DAT). Eventually, 1.5% NaCl was selected to be the ideal concentration for use in following survey. In addition, we decided to expose soybean plants to 3 mM cadmium (Cd), lead (Pb) and nickel (Ni) stresses.

### 4.2. Selection of the Appropriate Ampelopsin (AMP) Concentration

The seeds were surface sterilized and tested for viability as stated by a formerly explained method [130,131]. Plants were grown in a climate chamber under the above mentioned conditions. Soybean seedlings were haphazardly split into five groups: (i) control, irrigated with only distilled water (50 mL/plant); (ii) salinity treatment, irrigated with 1.5% NaCl (50 mL/plant); (iii) cadmium treatment, irrigated with 3 mM Cd (50 mL/plant); (iv) lead treatment, irrigated with 3 mM Pb (50 mL/plant); and (v) nickel treatment, irrigated with 3 mM Ni (50 mL/plant). Each treatment contained five replicates. Each group was exposed to the varied treatments for 7 d. Later, the salinity and heavy metal damaged seedlings were treated with 0, 0.1, 1, 5, 10 and 25 μM ampelopsin (50 mL/plant, once a day) on their roots for 7 d. The ampelopsin (Sigma-Aldrich, St. Louis, MO, USA) solution (25 μM, stock) was made by dissolving the solute in ethanol, and then dilution with distilled water to develop varying concentrations. Several plant growth characteristics were measured in plants of each concentration after 7 days of treatment. Moreover, 1 μM ampelopsin (AMP) concentration was found to be the perfect concentration for further investigation.

### 4.3. Impact of Exogenous Ampelopsin on Salinity and Heavy Metal-Damaged Soybean Seedlings

#### 4.3.1. Plant Preparation and Experimental Design

The soybean seedlings were grown under natural sunlight in a greenhouse, with 43% relative humidity and 24 °C/11 °C (day/night) temperature, and the seedlings were well watered daily. Identical seedlings (one seedling/pot) were picked after three weeks and then shifted to pots (10 cm × 10 cm). Experiments were carried out with three-week old soybean seedlings. They were split into six groups: the control group, irrigated with distilled water (50 mL/pot); the control group, irrigated with 1 μM AMP (50 mL/pot); the salinity group, irrigated with 1.5% NaCl (50 mL/pot); the cadmium group, irrigated with 3 mM Cd (50 mL/pot); the lead group, irrigated with 3 mM Pb (50 mL/pot); and the nickel group, irrigated with 3 mM Ni (50 mL/pot). Each group was treated for 12 days, after which the groups of unstressed and stressed seedlings were subdivided into two groups comprising an equal number of seedlings; this produced ten experimental groups, which are explained in Table 3. Each treatment was performed with five replicates. The soybean seedlings were regularly irrigated with AMP for 12 days and taken at the selected time point. The elite soybean seedlings were either instantly used or swiftly deactivated in liquid nitrogen and stocked at −80 °C. Soil moisture (70%), pH (~7), and EC (≤1.2) were noted prior the survey; eventually, soil samples from each pot (300 g/pot) of each treatment were inspected to ascertain moisture, pH, and EC employing a humidity tester (Model DM-5; Takemura Electric Works, Ltd., Tokyo, Japan) and conductivity meter (YSI Model 32, Yellow Spring, OH, USA) (Table S1).

#### 4.3.2. Growth Analysis and Chlorophyll Index Contents

Growth analysis was performed on the soybean seedlings by measuring various agronomic traits to examine the impact of each treatment on the seedlings. These traits involved plant height, root length, stem diameter, leaf area, plant fresh/dry weight, root fresh/dry weight and the number of leaves, all of which were noted at 12 days. Stem diameter and leaf area (leaf length/width) were measured with a digital Vernier caliper and a ruler. Plant height and root length were measured with a tape meter. In an initial salt and AMP screening experiment, chlorophyll concentration in leaves was estimated employing a SPAD meter (SPAD-502, Konica Minolta, Tokyo, Japan). The plant and root dry weight were assessed by oven drying them at 60 °C for 48 h [132].

#### 4.3.3. Chlorophyll a (Chl a), Chlorophyll b (Chl b), and Carotenoid Content

Chemically extracted pigments, Chla/Chlb, and carotenoid quantity were examined by spectrophotometric analysis [133]. About 100 mg of freshly harvested leaf were immersed in dimethyl sulfoxide (5 mL DMSO) and maintained at 65 °C for 3 h. The absorbance of leaf–DMSO aliquot was then recorded spectrophotometrically at selected wavelengths (Thermo Fisher Scientific, Waltham, MA, USA).

#### 4.3.4. Phytohormones: Abscisic Acid (ABA) and Salicylic Acid (SA)

ABA content of soybean plants was quantified based on formerly claimed procedures [134,135]. Nitrogen gas (N_2_) was used to desiccate the obtained extract, and methylation was carried out by adding diazomethane (CH_2_N_2_). Gas chromatography–mass spectrometry (Agilent 6890N Gas Chromatograph, Santa Clara, CA, USA) was employed to estimate ABA quantity. ThermoQuset software (Manchester, UK) was employed to detect reactions to ions (m/e of 162 and 190 for Me-ABA and 166 and 194 for Me-[^2^H_6_]-ABA).

To evaluate SA content in soybean plants, freeze dried sample (0.1 g) was used, as stated earlier [136,137]. Concisely, methanol (90% and 100%) was utilized to obtain extraction from freeze dried sample and then it was centrifuged (12,000 rpm, 15 min, 4 °C). The collected extract was desiccated and the dried remnant was dissolved in 5% trichloroacetic acid, followed by centrifugation (10,000 rpm, 10 min, 4 °C). Ethyl acetate:cyclopentane:isopropanol (49.5:49.5:1.0 ratio, *v*/*v*) was used to partition the supernatant. The topmost layer of solution was desiccated and consumed for SA quantification via high performance liquid chromatography (HPLC).

#### 4.3.5. Estimation of Amino Acid Content

The procedure claimed formerly was used to ascertain amino acid content [138]. Fifty milligrams of freeze dried powder samples was hydrolyzed with 1 mL hydrochloric acid (6N HCl, 24 h, 110 °C). The derivation was then evaporated and condensed under vacuum (80 °C, 24 h). The condensed remnant was then dissolved with 1 mL hydrochloric acid (0.02N HCl) and passed through a filter membrane (0.45-μm). At the end, the solution was loaded in Amino Acid analyzer (Hitachi High-Technologies Corporation, Tokyo, Japan).

#### 4.3.6. Quantification of Protein and Sugar Content

Previously claimed method [139] was used to assess protein content in the diverse treatments. The freeze dried powder samples (0.1 g) were blended with a 1 mL phosphate buffer (50 mM, pH 7.0) and then centrifuged (10,000 rpm, 10 min, 4 °C). The collected supernatant was treated with a proper reagent and its absorbance was evaluated at the chosen wavelength (595 nm). 

The quantity of sugar was deciphered as stated in former report [140]. Concisely, freeze dried samples were powdered, and derived with 80% ethanol. After this, the mixture was vacuum dried and the desiccated remnant was dissolved in deionized water (1 mL). The collected residues were passed through membrane filter (0.45 µm), and then loaded into a HPLC system (Millipore Co., Waters Chromatography, Milford, MA, USA).

#### 4.3.7. Assessment of Antioxidant Activity

The performance of polyphenol oxidase (PPO) and peroxidase (POD) was assayed according to the method stated by Putter [141]. Catalase (CAT) and superoxide dismutase (SOD) activities were explored via the previously described method [142,143]. To evaluate flavonoids, DPPH radical scavenging performances, and total polyphenols, specimens were inspected pursuing formerly mentioned procedures [143,144,145,146]. The mixture absorbance was quantified at preferred wavelengths employing a spectrophotometer (Thermo Fisher Scientific, Waltham, MA, USA).

#### 4.3.8. Hydrogen Peroxide (H_2_O_2_), Lipid Peroxidation and Fatty Acid

H_2_O_2_ amounts were deciphered as previously explained [147]. Freeze dried samples were crushed and, then, the powdered sample (0.3 g) was homogenized in ice bath with 5 mL 0.1% trichloroacetic acid (TCA). The homogenate was centrifuged at 12,000 rpm for 20 min. The supernatant (0.5 mL) was combined with 10 mM potassium phosphate buffer (0.5 mL, pH 7.0) and 1 M potassium iodide (1 mL). The mixture was stored at 25 °C for 20 min and its intensity was recorded at 390 nm using a spectrophotometer (Thermo Fisher Scientific, Waltham, MA, USA).

The thiobarbituric acid (TBA) test was used to estimate lipid peroxidation in leaves [148]. The reaction mixture comprised 0.5 mL of 0.1% TCA extract that was added to 1 mL of 0.5% TBA (prepared in 20% TCA). The mixture was incubated in boiling water (95 °C, 30 min), followed by cooling in ice bath (10 min). Afterwards, the mixture was centrifuged (12,000 rpm, 5 min) and the supernatant absorbance was read at 532 and 600 nm (T60 UV-Vis, PG Instruments Ltd., Wibtoft, UK).

Plant materials were analyzed for their fatty acids content with reference to a formerly published method [149]. Gas chromatography–mass spectrometry analysis was performed on an Agilent Model 7890A series (Agilent, Dover, DE, USA).

### 4.4. Gene Expression Analysis

Previously described method [150] was employed for total RNA preparation, cDNA synthesis and quantitative PCR (qPCR) assay from the soybean leaves at 12 days. For each sample, one μg of total RNA was employed to generate cDNA employing a BioFACT RT-Kit (BIOFACT, Daejeon, Korea) pursuing the producer’s conventional instruction. The generated cDNA was utilized in qRT–PCR reaction to quantify relative expression of candidate genes with an Illumina Eco system (Illumina, San Diego, CA, USA). Primer sequences utilized in this survey are represented in Supplemental Table S2.

### 4.5. Statistical Analysis

Five biological replicates were employed for treatment and control. SAS statistical software (version 9.4, SAS Institute, Cary, NC, USA) was utilized to compare and analyze the data through ANOVA. A Tukey test (*p* < 0.05) was employed to specify meaningful contradictions between treatments. Microsoft Excel 2017 was used to estimate the mean and standard deviation. Graphs were illustrated using Origin Pro (version 9.85, Origin Lab Corporation, Northampton, MA, USA).

## 5. Conclusions

The use of ampelopsin (AMP) not only magnified soybean vitality under salinity and heavy toxicity, but also certainly impelled soybean resistance to these abiotic stresses. The AMP, under abiotic caused stress, modulated host growth through mitigating salt and heavy metal augmentation in soybean plant. Additionally, AMP implementation changed host biochemistry to diminish the deleterious impacts of abiotic stresses. In the present study, salt and heavy metal stresses repressed many genes, while AMP was able to tackle the suppression effect of salt and heavy metal stresses and reawakened various suppressed genes. AMP also stimulated the expression of stress related genes, namely, *GmNAC109*, *GmFDL19*, *GmSAP16*, *GmWRKY12*, *GmWRKY142*, *GmERF75*, *GmFAD3*, and *GmAPX*. Altogether, the obtained results provided distinct proof to confirm the stress-soothing impact of AMP and suggest its potential application in agriculture.

## Figures and Tables

**Table 1:** Effect of ampelopsin application on soybean plant growth, chlorophyll a (Chla), chlorophyll b (Chlb), total chlorophyll (total Chl), and carotenoid contents under normal and stress conditions after 12 days of treatment (12DAT).

Treatment	Plant Height	Root Length	Stem Diameter	Leaf Length	Leaf Width	Plant Fresh Weight	Plant Dry Weight	Root Fresh Weight	Root Dry Weight	Chla	Chlb	Total Chl	Carotenoid	No. Leaf
	(cm)	(cm)	(cm)	(cm)	(cm)	(g)	(g)	(g)	(g)	µg/g FW	µg/g FW	(µg/g FW)	µg/g FW	
12DAT														
Cont	24.33 ± 0.16 b	25.5 ± 0.25 b	0.25 ± 0.02 ab	7.78 ± 0.11 a	5.12 ± 0.56 ab	5.77 ± 0.11 b	0.7 ± 0.05 b	3.06 ± 0.53 ab	0.17 ± 0.0 ab	28.88 ± 0.44 a	32.79 ± 2.3 ab	140.63 ± 0.31 ab	3.78 ± 0.11 ab	15.67 ± 0.16 ab
AMP	28.33 ± 1.16 a	31.17 ± 0.41 a	0.27 ± 0.03 a	8.44 ± 0.78 a	6.3 ± 0.15 a	8.82 ± 0.59 a	1.04 ± 0.02 a	3.45 ± 0.27 a	0.21 ± 0.0 a	29.2 ± 0.6 a	35.18 ± 2.41 a	144.45 ± 3.77 a	3.93 ± 0.03 a	17 ± 0.66 a
S	8.83 ± 0.58 g	15 ± 0.5 e	0.2 ± 0.0 b	3.91 ± 0.04 c	3.17 ± 0.41 c	2.13 ± 0.06 d	0.45 ± 0.02 de	1.5 ± 0.25 cde	0.1 ± 0.0 bc	18.72 ± 0.64 b	13.89 ± 0.05 h	82.88 ± 8.56 e	1.79 ± 0.1 d	12.67 ± 0.66 cd
S + AMP	19 ± 0.5 cd	23.5 ± 0.25 c	0.24 ± 0.02 ab	7.43 ± 0.28 a	4.94 ± 0.47 b	5.72 ± 0.14 b	0.69 ± 0.05 b	2.21 ± 0.1 bcd	0.15 ± 0.02	29.19 ± 0.59 a	20.41 ± 0.2 fg	127.86 ± 0.93 cd	3.05 ± 0.47 abc	14.67 ± 0.66 bc
Cd	14 ± 1.0 e	13.67 ± 0.16 e	0.21 ± 0.0 ab	4.26 ± 0.87 bc	3.11 ± 0.44 c	3.35 ± 0.32 c	0.32 ± 0.04 ef	1.33 ± 0.03 de	0.1 ± 0.0 c	28.86 ± 0.43 a	18.86 ± 0.43 g	124.91 ± 3.54 d	2.96 ± 0.48 bc	12.33 ± 0.83 de
Cd + AMP	19 ± 0.5 cd	24 ± 1.0 bc	0.24 ± 0.02 ab	7.46 ± 0.27 a	4.8 ± 0.4 b	5.7 ± 0.15 b	0.68 ± 0.06 b	3.03 ± 0.48 ab	0.16 ± 0.02 b	29.43 ± 0.71 a	26.25 ± 1.12 de	135.27 ± 4.63 abcd	3.77 ± 0.11 ab	15.33 ± 0.33 ab
Pb	11.67 ± 0.16 f	14.17 ± 0.91 e	0.21 ± 0.0 ab	5.45 ± 0.27 b	3.38 ± 0.31 c	5.03 ± 0.51 b	0.53 ± 0.06 cd	1.42 ± 0.29 cde	0.08 ± 0.01 cd	29.2 ± 0.6 a	22.78 ± 0.61 ef	132.23 ± 3.11 bcd	3.11 ± 0.44 abc	11.67 ± 0.16 de
Pb + AMP	18 ± 0.5 d	21.17 ± 0.41 d	0.25 ± 0.02 ab	7.62 ± 0.19 a	4.86 ± 0.43 b	5.77 ± 0.11 b	0.7 ± 0.05 b	2.66 ± 0.33 ab	0.16 ± 0.02 b	29.71 ± 0.85 a	27.64 ± 0.82 cd	137.84 ± 1.92 abc	3.78 ± 0.11 ab	15.33 ± 0.33 ab
Ni	13.17 ± 0.41 ef	10.5 ± 0.75 f	0.2 ± 0.0 b	4.57 ± 0.71 bc	2.6 ± 0.3 c	2.38 ± 0.19 d	0.29 ± 0.04 f	0.67 ± 0.06 e	0.05 ± 0.0 d	29.67 ± 0.83 a	24.49 ± 0.24 de	134.17 ± 0.91 abcd	2.81 ± 0.4 c	10.33 ± 0.83 e
Ni + AMP	20.67 ± 0.66 c	22.33 ± 0.83 cd	0.25 ± 0.02 ab	7.58 ± 0.21 a	4.95 ± 0.47 b	5.57 ± 0.21 b	0.63 ± 0.01 bc	2.27 ± 0.13 bc	0.15 ± 0.02 b	29.45 ± 0.72 a	30.64 ± 0.32 bc	140.25 ± 0.12 ab	3.77 ± 0.11 ab	15.67 ± 1.16 ab

Treatments: Cont (control), AMP (1 μM ampelopsin), S (1.5 % sodium chloride), AMP (1 μM ampelopsin) + S (1.5 % sodium chloride), Cd (3 mM cadmium), 1 μM AMP (ampelopsin) + Cd (3 mM cadmium), Pb (3 mM lead), AMP (1 μM ampelopsin) + Pb (3 mM lead), Ni (3 mM nickel), and AMP (1 μM ampelopsin) + Ni (3 mM nickel). Values show the means ± SE (*n* = 5) and significant differences at *p* < 0.05 (Tukey test). Data within the same column followed by different lowercase letters are significantly different.

**Table 2:** Effect of ampelopsin application on the amino acid content of soybean plants grown under normal and stress conditions, after 12 days of treatment (12DAT).

Amino Acid	Treatment									
mg/g	Cont	AMP	S	S + AMP	Cd	Cd + AMP	Pb	Pb + AMP	Ni	Ni + AMP
12DAT										
Asp	32.96 ± 0.48 h	46.53 ± 0.26 f	58.32 ± 0.16 c	67.04 ± 0.52 a	48.04 ± 0.02 e	60.29 ± 0.14 b	56.22 ± 0.11 d	61.23 ± 0.61 b	39.27 ± 0.63 g	46.41 ± 0.20 f
Thr	16.2 ± 0.1 f	18.35 ± 0.17 e	21.15 ± 0.57 bcd	22.24 ± 0.12 ab	20.5 ± 0.25 d	20.83 ± 0.41 cd	22.01 ± 0.0 abc	23.25 ± 0.62 a	18.18 ± 0.09 e	21.46 ± 0.73 bcd
Ser	15.02 ± 0.51 e	17.07 ± 0.53 d	22.58 ± 0.29 b	29.78 ± 0.89 a	19.57 ± 0.78 c	19.74 ± 0.87 c	20.79 ± 0.39 bc	21.4 ± 0.7 bc	16.57 ± 0.28 de	20.05 ± 0.02 c
Glu	40.2 ± 0.1 e	46.22 ± 0.11 d	46.7 ± 0.35 d	55.24 ± 2.62 ab	52.05 ± 0.02 c	53.09 ± 0.54 bc	52.02 ± 0.01 c	57.34 ± 0.67 a	45.37 ± 0.68 d	52.31 ± 0.15 c
Gly	18.39 ± 0.19 e	20.48 ± 0.24 d	21.2 ± 0.6 d	24.22 ± 0.11 b	22.5 ± 0.25 c	23.38 ± 0.69 bc	24.33 ± 0.16 b	26.15 ± 0.07 a	20.55 ± 0.27 d	24.13 ± 0.06 b
Ala	21.66 ± 0.83 e	24.06 ± 0.03 d	26.46 ± 0.23 c	28.57 ± 0.28 b	26.39 ± 0.19 c	27.39 ± 0.69 bc	28.53 ± 0.26 b	30.59 ± 0.29 a	24.14 ± 0.07 d	28.25 ± 0.12 b
Cys	1.66 ± 0.17 de	2.19 ± 0.09 cde	2.22 ± 0.11 bcde	3.02 ± 0.51 abc	3.18 ± 0.41 a	3.28 ± 0.36 a	1.55 ± 0.22 e	2.46 ± 0.23 abcde	2.52 ± 0.06 abcd	3.16 ± 0.42 ab
Val	19.22 ± 0.61 d	21.47 ± 0.73 c	24.14 ± 0.07 b	25.54 ± 0.77 ab	24.13 ± 0.06 b	25.23 ± 0.61 b	25.29 ± 0.64 b	27.35 ± 0.67 a	21.66 ± 0.83 c	24.71 ± 0.35 b
Met	1.57 ± 0.21 c	2.32 ± 0.16 abc	2.54 ± 0.27 ab	2.6 ± 0.3 ab	2.14 ± 0.07 bc	2.44 ± 0.22 ab	2.21 ± 0.1 bc	3.02 ± 0.51 a	2.30 ± 0.15 abc	2.31 ± 0.15 abc
Ile	15.56 ± 0.78 e	17.69 ± 0.84 d	19.2 ± 0.6 bcd	20.7 ± 0.35 b	19.59 ± 0.79 bc	20.62 ± 0.31 b	20.57 ± 0.28 b	22.46 ± 0.23 a	18.1 ± 0.05 cd	20.43 ± 0.21 b
Leu	33.58 ± 0.79 e	37.67 ± 0.83 d	39.12 ± 0.56 d	44.45 ± 0.22 b	41.22 ± 0.61 c	43.05 ± 0.52 b	44.23 ± 0.11 b	48.05 ± 0.02 a	37.66 ± 0.83 d	44.14 ± 0.07 b
Tyr	9.31 ± 0.34 c	10.37 ± 0.81 bc	11.1 ± 0.45 b	13.4 ± 0.3 a	13.14 ± 0.43 a	13.23 ± 0.38 a	11.02 ± 0.49 b	14.11 ± 0.055 a	11.36 ± 0.32 b	13.37 ± 0.31 a
Phe	18.6 ± 0.7 e	21.22 ± 0.61 d	23.46 ± 0.73 bc	25.06 ± 0.53 b	23.24 ± 0.62 c	24.13 ± 0.06 bc	24.28 ± 0.14 bc	27.03 ± 0.51 a	21.28 ± 0.64 d	24.53 ± 0.26 bc
Lys	25.49 ± 0.74 e	28.37 ± 0.18 d	29.19 ± 0.59 d	34.05 ± 0.02 ab	31.65 ± 0.82 c	33.31 ± 0.65 bc	32.52 ± 0.26 bc	35.63 ± 0.18 a	28.16 ± 0.92 d	33.39 ± 0.69 bc
NH3	4.06 ± 0.03 e	6.58 ± 0.29 c	8.51 ± 0.28 b	11.68 ± 0.84 a	6.03 ± 0.01 cd	8.08 ± 0.04 b	8.56 ± 0.28 b	11.47 ± 0.73 a	5.06 ± 0.53 de	5.44 ± 0.28 cd
His	8.09 ± 0.04 d	9.53 ± 0.76 bcd	11.54 ± 0.77 a	11.61 ± 0.19 a	10.68 ± 0.34 abc	11.06 ± 0.53 ab	11.47 ± 0.73 a	12.21 ± 0.1 a	9.21 ± 0.6 cd	10.67 ± 0.33 abc
Arg	16.65 ± 0.32 g	19.07 ± 0.53 f	23.63 ± 0.81 bc	26.16 ± 0.08 a	20.6 ± 0.3 e	21.6 ± 0.2 de	22.6 ± 0.0 cd	24.29 ± 0.14 b	19.2 ± 0.6 f	22.54 ± 0.27 cd
Pro	13.64 ± 0.82 d	14.66 ± 0.67 cd	16.35 ± 0.17 bc	17.17 ± 0.58 b	16.15 ± 0.07 bc	17.26 ± 0.63 b	16.44 ± 0.22 b	19.39 ± 0.69 a	16.0 ± 0.5 bc	17.02 ± 0.51 b

Treatments: Cont (control), AMP (1 μM ampelopsin), S (1.5% sodium chloride), AMP (1 μM ampelopsin) + S (1.5% sodium chloride), Cd (3 mM cadmium), 1 μM AMP (ampelopsin) + Cd (3 mM cadmium), Pb (3 mM lead), AMP (1 μM ampelopsin) + Pb (3 mM lead), Ni (3 mM nickel), and AMP (1 μM ampelopsin) + Ni (3 mM nickel). Values show the means ± SE (*n* = 5) and significant differences at *p* < 0.05 (Tukey test). Data within the same row followed by different lowercase letters are significantly different.

**Table 3:** Experimental work plan.

Symbol	Treatment
Cont	irrigated with sterile distilled water
AMP	irrigated with 1 μM AMP
S	irrigated with 1.5% NaCl
S + AMP	irrigated with 1.5% NaCl + 1 μM AMP
Cd	irrigated with 3 mM Cd
Cd + AMP	irrigated with 3 mM Cd + 1 μM AMP
Pb	irrigated with 3 mM Pb
Pb + AMP	irrigated with 3 mM Pb + 1 μM AMP
Ni	irrigated with 3 mM Ni
Ni + AMP	irrigated with 3 mM Ni + 1 μM AMP

## Data Availability

The data presented in this study are available in the tables and Supplementary Tables.

## Supplementary Materials

The following are available online at https://www.mdpi.com/article/10.3390/ijms222010943/s1.
