# Supplementary material for: Ampelopsin Confers Endurance and Rehabilitation Mechanisms in Glycine max cv. Sowonkong under Multiple Abiotic Stresses
Source: Int J Mol Sci. 2021 Oct 10;22(20):10943. doi: 10.3390/ijms222010943 (PMC8536110; doi:10.3390/ijms222010943)
Supplement: Supplementary file 1 [file ijms-22-10943-s001.zip › ijms-1370327-supplementary.pdf]

Supplementary materials

Ampelopsin confers endurance and rehabilitation mechanisms in *Glycine max* cv. Sowonkong under multiple abiotic stresses

Elham Ahmed Kazerooni[1*], Abdullah Mohammed Al-Sadi[2], Il-Doo Kim[1], Muhammad Imran[1], In-Jung Lee[1*]

[1]Department of Applied Biosciences, Kyungpook National University, Daegu 41566, Republic of Korea

[2]Department of Plant Sciences, College of Agricultural and Marine Sciences, Sultan Qaboos University, PO Box 34, Al-Khod 123, Oman

[*]Corresponding authors

Prof. In-Jung lee
Dr. Elham Ahmed Kazerooni
Crop Physiology Laboratory
Department of Applied Biosciences
Kyungpook National University
Daegu 41566, Republic of Korea
E-mail: ijlee@knu.ac.kr
E-mail: elham.ghasemi.k@gmail.com
Tel: +82-53-950-5708(Office)

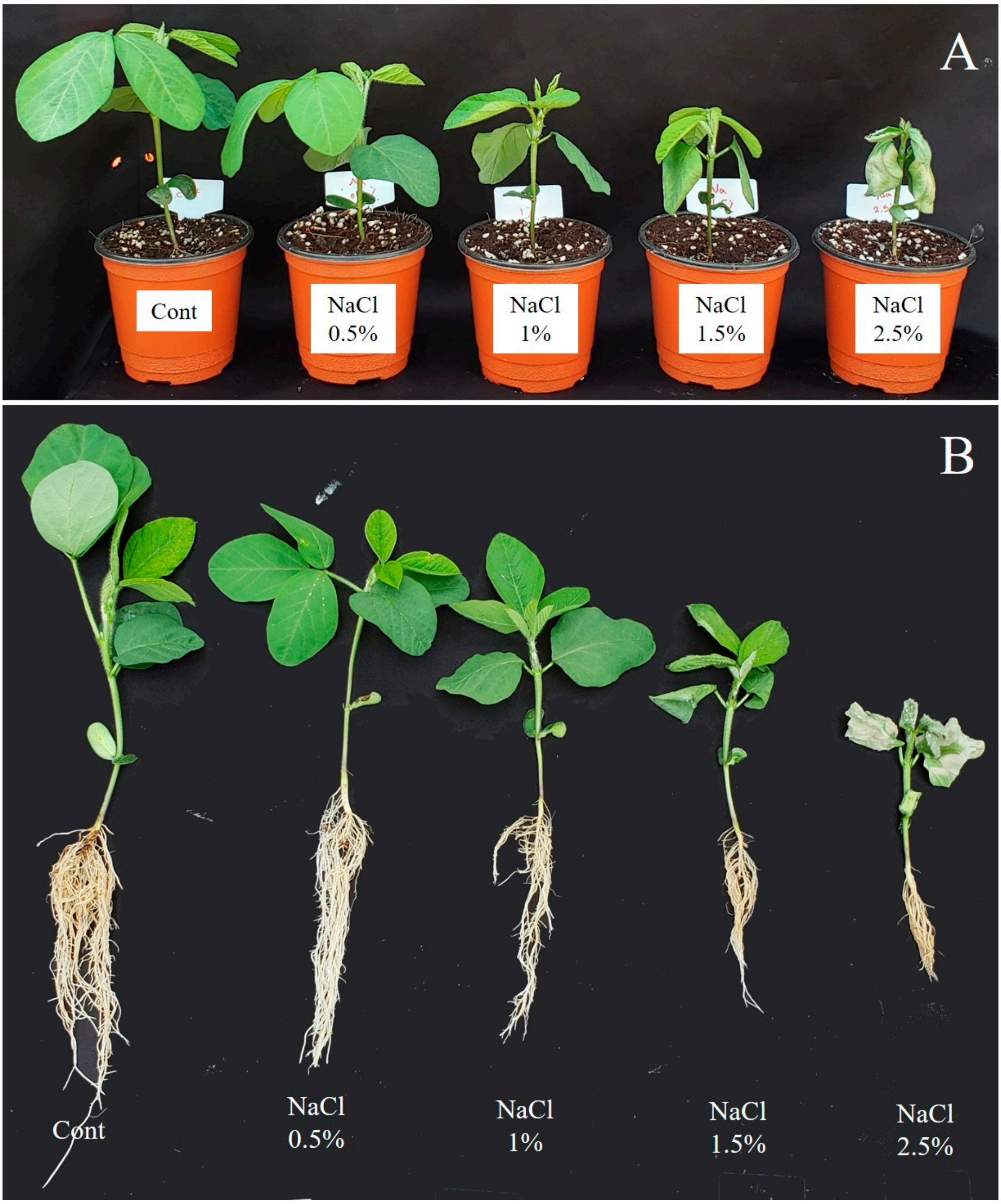

Figure S1. Effects of various salt (NaCl) concentrations on the growth of soybean seedlings following 7 days of treatment (7DAT; A and B). Treatments: Cont (control), 0.5% NaCl, 1% NaCl, 1.5% NaCl, and 2.5% NaCl.

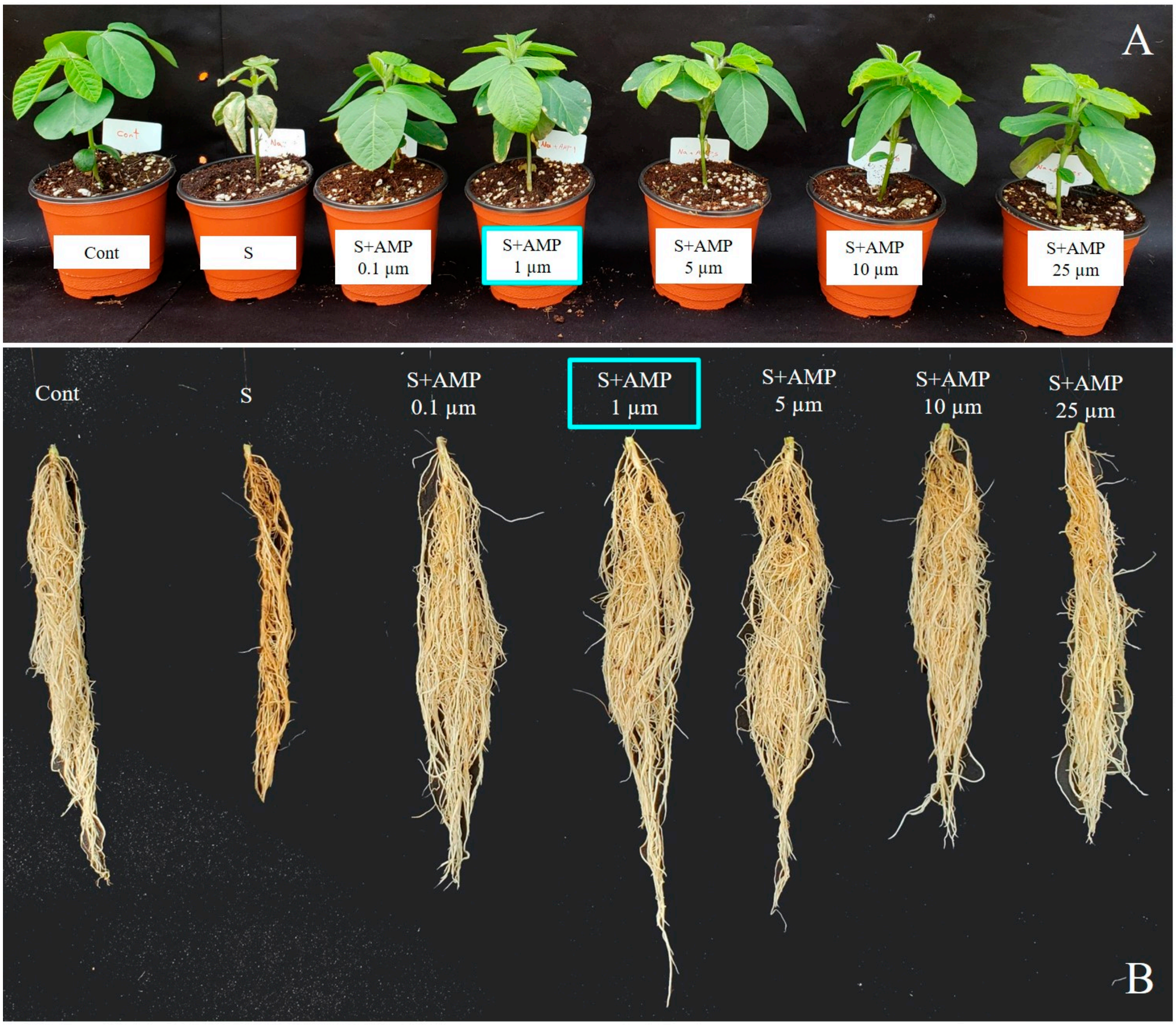

Figure S2. Effects of various ampelopsin concentrations on the growth of stressed-soybean seedlings following 7 days of sodium chloride treatment (7DAT; A and B). Treatments: Cont (control), 1.5% NaCl (sodium chloride), 0.1 µM AMP (ampelopsin) + 1.5% NaCl (sodium chloride), 1 µM AMP (ampelopsin) + 1.5% NaCl (sodium chloride), 5 µM AMP (ampelopsin) + 1.5% NaCl (sodium chloride), 10 µM AMP (ampelopsin) + 1.5% NaCl (sodium chloride), and 25 µM AMP (ampelopsin) + 1.5% NaCl (sodium chloride).

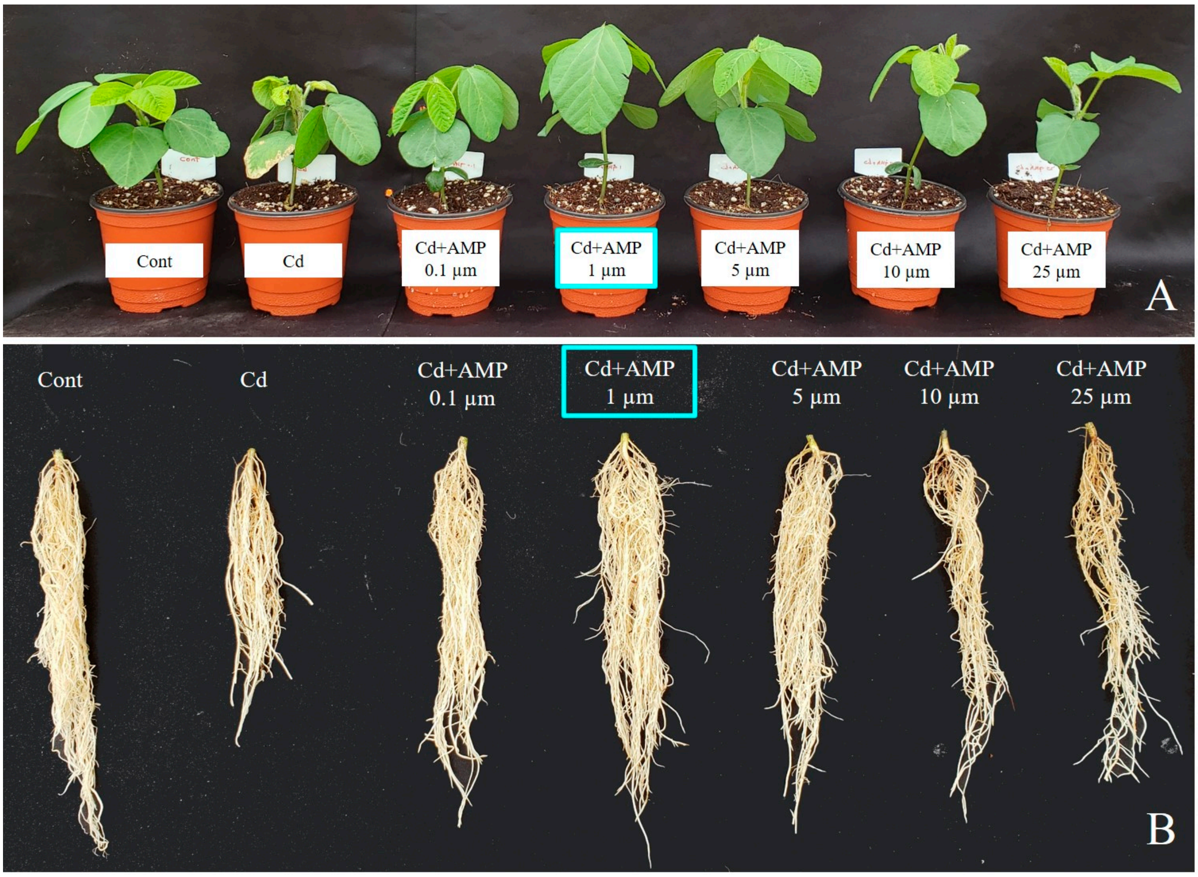

Figure S3. Effects of various ampelopsin concentrations on the growth of soybean seedlings following 7 days of cadmium treatment (7DAT; A and B). Treatments: Cont (control), 3mM Cd (cadmium), 0.1 µM AMP (ampelopsin) + 3mM Cd (cadmium), 1 µM AMP (ampelopsin) + 3mM Cd (cadmium), 5 µM AMP (ampelopsin) + 3mM Cd (cadmium), 10 µM AMP (ampelopsin) + 3mM Cd (cadmium), and 25 µM AMP (ampelopsin) + 3mM Cd (cadmium).

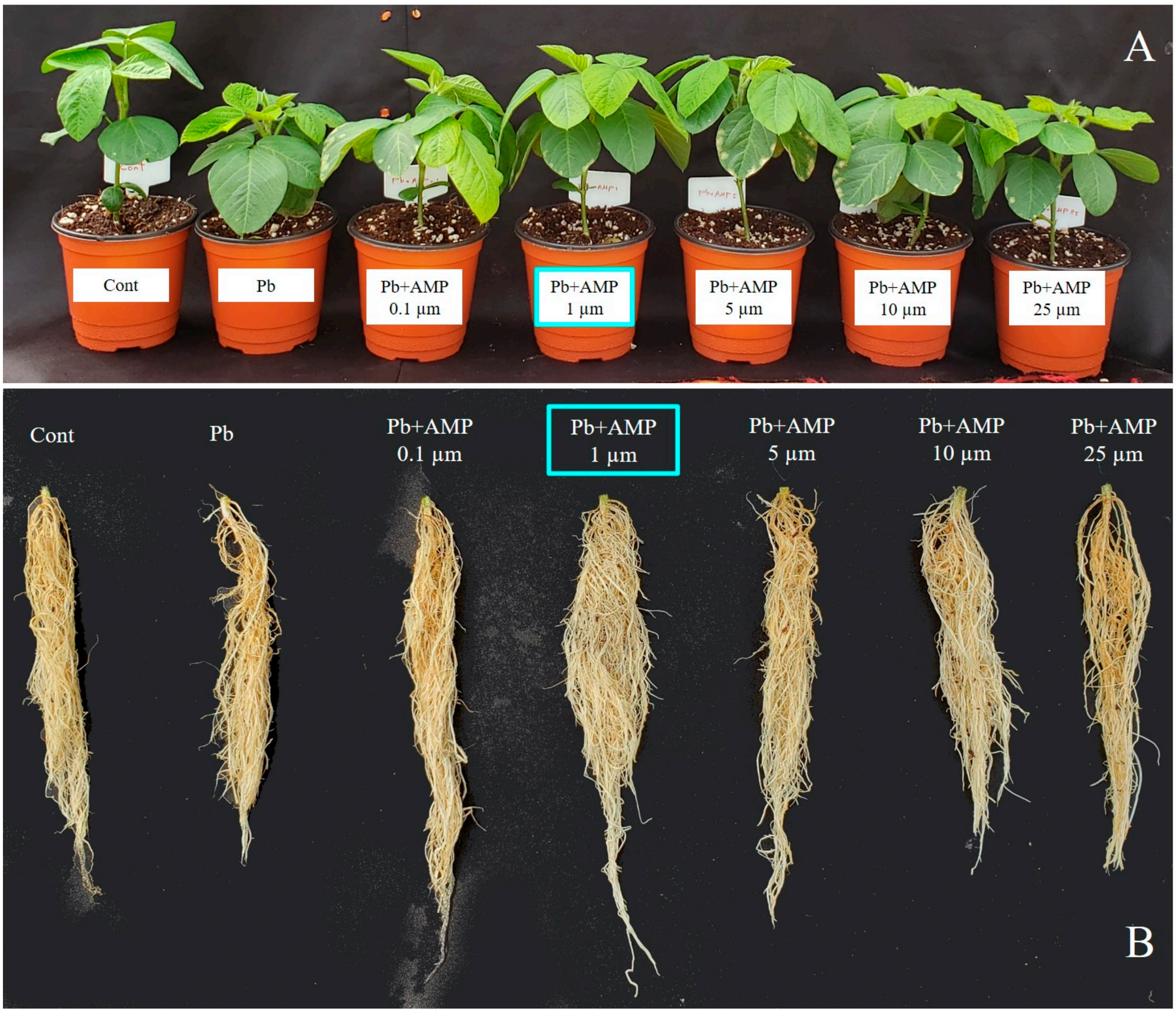

Figure S4. Effects of various ampelopsin concentrations on the growth of soybean seedlings following 7 days of lead treatment (7DAT; A and B). Treatments: Cont (control), 3mM Pb (lead), 0.1 µM AMP (ampelopsin) + 3mM Pb (lead), 1 µM AMP (ampelopsin) + 3mM Pb (lead), 5 µM AMP (ampelopsin) + 3mM Pb (lead), 10 µM AMP (ampelopsin) + 3mM Pb (lead), and 25 µM AMP (ampelopsin) + 3mM Pb (lead).

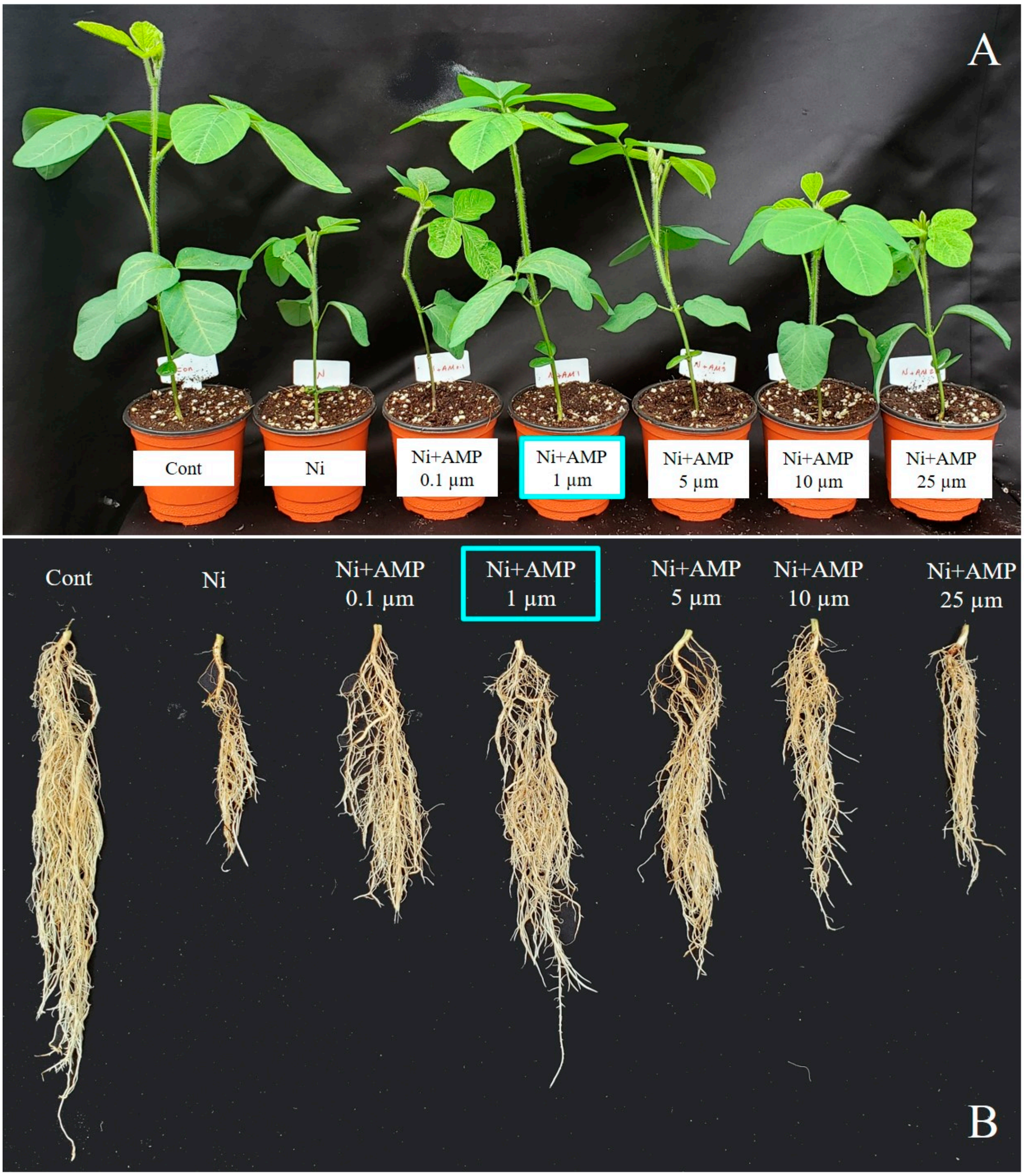

Figure S5. Effects of various ampelopsin concentrations on the growth of soybean seedlings following 12 days of nickel treatment (12DAT; A and B). Treatments: Cont (control), 3mM Ni (nickel), 0.1 µM AMP (ampelopsin) + 3mM Ni (nickel), 1 µM AMP (ampelopsin) + 3mM Ni (nickel), 5 µM AMP (ampelopsin) + 3mM Ni (nickel), 10 µM AMP (ampelopsin) + 3mM Ni (nickel), and 25 µM AMP (ampelopsin) + 3mM Ni (nickel).

Table S1. Physiochemical properties of soil samples during 12 days of treatment (12DAT).

Sample name	Soil texture	pH	EC (mS)	Moisture (%)
12DAT				
Cont	sandy loam	7.15±0.05b	0.33±0.01j	70.7±0.1g
AMP	sandy loam	8.17±0.03a	0.59±0.01i	77.73±0.07f
S	sandy loam	6.5±0.05e	11.81±0.8a	100.0±0.0a
S+AMP	sandy loam	7.05±0.05c	6.34±0.01b	94.7±0.3e
Cd	sandy loam	5.05±0.05i	5.75±0.01c	100.0±0.0a
Cd+AMP	sandy loam	6.05±0.05f	2.53±0.03f	97.8±0.2b
Pb	sandy loam	5.6±0.1g	5.63±0.08	100.0±0.0a
Pb+AMP	sandy loam	6.95±0.05d	2.24±0.02h	95.1±0.1d
Ni	sandy loam	5.4±0.1h	5.65±0.08	100.0±0.0a
Ni+AMP	sandy loam	6.05±0.05f	2.33±0.03g	96.8±0.2c

Values show the means ± SE (n = 5) and significant differences at $p < 0.05$ (LSD test). Data within the same column followed by different lowercase letters are significantly different.

Table S2. Primers used for relative gene expression analysis.

Gene symbol	Primers (5′–3′) Forward/reverse
GmNAC109	5′-CACCATGGCCACTACAACACAAC-3′<br>5′-TTAATTGCAGAAGGACTTGGAGA-3′
GmWRKY12	5′-CAATACTGCTGTGGCCGCTA-3′<br>5′-ATCCAGTGTTCGCACCTGTT-3′
GmWRKY142	5′-TTATGGACAAAGGATGGGTT-3′<br>5′-GCCCTTGTCTATTTACTCCC-3′
GmERF75	5′-ATGGCGAACGCAGCTGAAGTTT-3′<br>5′-TCACACCGCCACGAGCG-3′
GmFAD3	5′-AAAACGCCTATGGTTAAAGACACAAAG-3′<br>5′-AAAAGGCCTGTGTCGTTGCGAGTGGAG-3′
GmSAP16	5′-CGATTTTCTTCCTTTCACC-3′<br>5′-GCTTCGCAAACAACAACCT-3′
GmFDL19	5′-GGTTTGGAGAGATATGCAAC-3′<br>5′-GGCATGTTGTGATGTGTTGT-3′
GmAPX	5′-ATCCTTGCTACGCACACACT-3′<br>5′-TCCACATGCTTGGTCCACA-3′
Actin	5′-TTGACTGAGCGTGGTTATTCC-3′<br>5′-GATCTTCATGCTGCTGGGTG-3′

Table S3. Effects of various salt (NaCl) concentrations on the growth of soybean seedlings. Data were calculated after 7 days of treatment (7DAT).

Treatment	Plant height (cm)	Root length (cm)	Stem diameter (cm)	Leaf length (cm)	Leaf width (cm)	Chl (Spad)	Plant fresh weight (g)	Plant dry weight (g)	Root fresh weight (g)	Root dry weight (g)	No. leaf
7DAT											
Cont	15.83±0.6a	21±0.58a	0.32±0.02a	7.67±0.28a	5.82±0.41a	48±0.46a	5.07±0.33a	0.54±0.01a	2.11±0.03a	0.15±0.01a	13±0.0a
NaCl (0.5%)	12.7±0.15b	15.5±0.29b	0.21±0.0b	5.79±0.03b	4.03±0.01b	31.77±0.38b	2.94±0.08b	0.36±0.01b	1.45±0.08b	0.09±0.01b	11±0.0b
NaCl (1%)	9.33±0.33c	13.5±0.29c	0.2±0.0c	4.92±0.19c	3.07±0.06c	29.37±0.52c	2.37±0.08c	0.3±0.01c	0.7±0.01c	0.03±0.0c	10±0.0c
NaCl (1.5%)	7.33±0.33d	9.83±0.6d	0.19±0.0d	3.71±0.12d	2.35±0.09d	21.33±0.88d	1.68±0.04d	0.25±0.01d	0.54±0.01d	0.02±0.0d	8.33±0.33d
NaCl (2.5%)	6.33±0.33e	5.9±0.31e	0.16±0.0e	2.2±0.06e	1.17±0.05e	10.77±0.34e	1.19±0.01e	0.24±0.01e	0.43±0.02e	0.01±0.0e	5.33±0.33e

Treatments: Cont (control), 0.5% NaCl, 1% NaCl, 1.5% NaCl, and 2.5% NaCl. Values show the means ± SE (n = 5) and significant differences at $p < 0.05$ (LSD test). Data within the same column followed by different lowercase letters are significantly different.

Table S4. Effects of various ampelopsin (AMP) concentrations on the growth of salinity (1.5% NaCl) and heavy metal (3mM Cd, Pb, and Ni) stressed soybean seedlings. Data were calculated after 7 days of treatment (7DAT).

Treatment	Plant height (cm)	Root length (cm)	Stem diameter (cm)	Leaf length (cm)	Leaf width (cm)	Chl (Spad)
7DAT						
Cont	10.25±0.25d	20.5±0.5c	0.23±0.0a	6.24±0.0c	4.06±0.01d	32.55±0.55f
S	7.5±0.5g	13.5±0.5g	0.17±0.0f	3.11±0.0g	2.93±0.04g	25.85±0.25g
S+AMP (0.1 µm)	10.5±0.5c	19.7±0.2d	0.18±0.0e	5.95±0.0d	4.56±0.0c	33.9±0.1e
S+AMP (1 µm)	13.5±0.5a	23.75±0.25a	0.22±0.0b	6.72±0.0a	4.67±0.0a	42.6±0.4a
S+AMP (5 µm)	11.5±0.5b	20.75±0.25b	0.20±0.0c	6.37±0.0b	4.63±0.02b	38.5±0.5b
S+AMP (10 µm)	9.5±0.5e	19.25±0.25e	0.19±0.0d	5.79±0.0e	3.91±0.0e	37.75±0.15c
S+AMP (25 µm)	8.5±0.5f	18.5±0.5f	0.18±0.0e	5.28±0.0f	3.90±0.0f	37.55±0.55d
Cont	10.25±0.25e	20.5±0.5c	0.23±0.0a	6.24±0.0b	4.06±0.01d	32.55±0.55c
Cd	8.5±0.5f	14±1.0g	0.18±0.02e	4.03±0.01g	2.91±0.0g	20.0±0.4g
Cd+AMP (0.1 µm)	10.75±0.25d	21.0±0.5b	0.19±0.0d	6.07±0.0d	4.25±0.05c	32.8±0.2b
Cd+AMP (1 µm)	14.75±0.75a	24.25±0.75a	0.23±0.0a	8.82±0.02a	6.5±0.0a	42.35±0.65a
Cd+AMP (5 µm)	12.25±0.25b	20.25±0.25d	0.21±0.0b	6.17±0.0c	4.58±0.0b	30.7±0.3d
Cd+AMP (10 µm)	12.0±0.0c	19.25±0.25e	0.20±0.0c	5.03±0.0e	3.26±0.0e	28.95±0.05e
Cd+AMP (25 µm)	10.75±0.25d	18.75±0.25f	0.20±0.0c	4.78±0.0f	2.97±0.0f	25.7±0.3f
Cont	10.25±0.25f	20.5±0.5a	0.23±0.0a	6.24±0.0c	4.06±0.01c	32.55±0.55a
Pb	7.5±0.5g	12.75±0.75g	0.18±0.0e	5.43±0.0f	3.30±0.02f	23.5±0.5g
Pb+AMP (0.1 µm)	11.25±0.25c	17.75±0.75e	0.19±0.0d	6.85±0.0b	4.56±0.0b	29.5±0.2c
Pb+AMP (1 µm)	14.5±0.5a	20.0±1.0b	0.22±0.0b	7.48±0.01a	5.08±0.0a	31.9±1.1b
Pb+AMP (5 µm)	12.5±0.5b	19.75±0.25c	0.20±0.0c	5.66±0.0d	3.48±0.0d	28.7±0.3d
Pb+AMP (10 µm)	11.0±0.0d	18.25±0.25d	0.19±0.0d	5.48±0.0e	3.40±0.0e	27.8±0.2e
Pb+AMP (25 µm)	10.5±0.0e	17.25±0.25f	0.19±0.0d	4.39±0.0g	3.16±0.0g	25.15±0.85f
Cont	10.25±0.25f	20.5±0.5b	0.23±0.0b	6.24±0.0c	4.06±0.01c	32.55±0.55b
Ni	9.1±0.72g	10.5±0.5g	0.19±0.01f	4.57±0.17f	2.60±0.15g	22.4±1.54g
Ni+AMP (0.1 µm)	11.3±0.33e	14.66±0.33e	0.21±0.0d	5.08±0.01e	3.67±0.02d	28.8±0.2d
Ni+AMP (1 µm)	14.33±0.33a	21.33±0.88a	0.24±0.0a	7.54±0.37a	4.81±0.14a	36.8±1.8a
Ni+AMP (5 µm)	13.33±0.33b	16.83±0.44c	0.23±0.0b	6.64±0.02b	4.75±0.01b	31.96±0.03c
Ni+AMP (10 µm)	12.33±0.33c	15.5±0.28d	0.22±0.0c	5.43±0.03d	3.53±0.03e	27.5±0.5e
Ni+AMP (25 µm)	11.33±0.33d	13.66±0.33f	0.20±0.0e	4.50±0.0g	3.17±0.02f	24.2±0.41f

Values show the means ± SE (n = 5) and significant differences at $p < 0.05$ (LSD test). Data within the same column followed by different lowercase letters are significantly different.
